# Laparoscopic right hemicolectomy with CME: standardization using the “critical view” concept

**DOI:** 10.1007/s00464-018-6267-0

**Published:** 2018-10-15

**Authors:** Christoph Werner Strey, Christoph Wullstein, Michel Adamina, Ayman Agha, Heiko Aselmann, Thomas Becker, Robert Grützmann, Werner Kneist, Matthias Maak, Benno Mann, Kurt Thomas Moesta, Norbert Runkel, Clemens Schafmayer, Andreas Türler, Thilo Wedel, Stefan Benz

**Affiliations:** 1 Clinic for General-, Visceral- and Vascular Surgery, Diakovere Hospital Friederikenstift, Humboldtstrasse 5, 30169 Hannover, Germany; 2 Department of Visceral and Minimal Invasive Surgery, Helios Hospital Krefeld, Lutherplatz 40, 47805 Krefeld, Germany; 3 Department Chirurgie, Klinik für Viszeral- und Thoraxchirurgie, Kantonsspital Winterthur, Brauerstrasse 15, Postfach 834, 8401 Winterthur, Switzerland; 4 Klinik für Allgemein-, Viszeral-, Endokrine und Minimal-invasive Chirurgie, Klinikum Bogenhausen, Englschalkinger Straße 77, 81925 Munich, Germany; 5 General and Visceral Surgery, DRK-Krankenhaus Clementinenhaus, Lützerodestr. 1, 30161 Hannover, Germany; 6 Klinik für Allgemeine, Viszeral-, Thorax-, Transplantations- und Kinderchirurgie, Universitätsklinikum Schleswig-Holstein, Arnold-Heller-Straße 3, 24105 Kiel, Germany; 7 Department of Surgery, University Hospital of Erlangen, Krankenhausstraße 12, 91054 Erlangen, Germany; 8 Department of General, Visceral and Transplant Surgery, University Medical Center, University of the Johannes Gutenberg-University Mainz, Langenbeckstraße 1, 55131 Mainz, Germany; 9 Klinik für Viszeralchirurgie, Augusta Klinikum Bochum, Bergstrasse 26, 44791 Bochum, Germany; 10 Department of General-, Visceral- and Minimalinvasive Surgery, KRH Klinikum Hannover GmbH, Klinikum Siloah, Stadionbrücke 4, 30459 Hannover, Germany; 11 Sana Klinikum, Starkenburgring 66, 63069 Offenbach, Germany; 12 Department of General Surgery and Thoracic Surgery, University Hospital Schleswig-Holstein, 24105 Kiel, Germany; 13 Department of Visceral Surgery, Johanniter Hospital Bonn, Johanniterstr. 3, 53113 Bonn, Germany; 14 Institute of Anatomy, Center of Clinical Anatomy, Christian-Albrechts University Kiel, Otto-Hahn-Platz 8, 24118 Kiel, Germany; 15 Department for Abdominal and Pediatric Surgery, Klinkverbund-Suedwest, Klinken Boeblingen, Bunsenstrasse 120, 71032 Boeblingen, Germany

**Keywords:** Colon cancer, Right hemicolectomy, Laparoscopy, Complete mesocolic excision, Critical view, Standardization

## Abstract

**Background:**

Complete mesocolic excision is gradually becoming an established oncologic surgical principle for right hemicolectomy. However, the procedure is technically demanding and carries the risk of serious complications, especially when performed laparoscopically. A standardized procedure that minimizes technical hazards and facilitates teaching is, therefore, highly desirable.

**Methods:**

An expert group of surgeons and one anatomist met three times. The initial aim was to achieve consensus about the surgical anatomy before agreeing on a sequence for dissection in laparoscopic CME. This proposal was evaluated and discussed in an anatomy workshop using post-mortem body donors along with videos of process-informed procedures, leading to a definite consensus.

**Results:**

In order to provide a clear picture of the surgical anatomy, the “open book” model was developed, consisting of symbolic pages representing the corresponding dissection planes (retroperitoneal, ileocolic, transverse mesocolic, and mesogastric), vascular relations, and radicality criteria. The description of the procedure is based on eight preparative milestones, which all serve as critical views of safety. The chosen sequence of the milestones was designed to maximize control during central vascular dissection. Failure to reach any of the critical views should alert the surgeon to a possible incorrect dissection and to consider converting to an open procedure.

**Conclusion:**

Combining the open-book anatomical model with a clearly structured dissection sequence, using critical views as safety checkpoints, may provide a safe and efficient platform for teaching laparoscopic right hemicolectomy with CME.

**Electronic supplementary material:**

The online version of this article (10.1007/s00464-018-6267-0) contains supplementary material, which is available to authorized users.

Precision of oncological resections has an impact on cancer recurrence and survival. In total mesorectal excision (TME) [1], the key quality factor for colon resections is the necessity to respect embryonic planes. Therefore, Hohenberger coined the term “Complete Mesocolic Excision” (CME) [2]. TME results in a higher number of resected lymph nodes with a corresponding reduction of recurrence rates [3, 4]. The evidence from cohort analyses on the oncological effects of CME [5, 6] have resulted in the CME concept becoming a standard in many centers, including the right hemicolectomy surgery. CME is included in the German guideline for treatment of colorectal cancer. The number of studies investigating the oncological effectiveness of CME has grown steadily [7]. However, there is ongoing debate concerning its safety and complication rate when compared to conventional colonic resections [8].

Before the CME era, laparoscopic colorectal procedures were viewed as oncological equivalent to open surgery [9]. To meet the requirements of CME, the laparoscopic technique that was initially described in hemicolectomy requires adaptation [10]. Different techniques of laparoscopic right hemicolectomy with CME have been proposed [11–14] but the complexity of this operation, in respect to vascular variability [15] is high, and the procedure bears a significant risk of complications. Before this procedure can be generally recommended, a consensus is needed on how the operation can be carried out optimally. In contrast to the open operation [16], this has not yet been achieved for current laparoscopic procedures [11–13].

The objective of our working group was to establish a standardized procedure for laparoscopic right hemicolectomy that meets all CME criteria, with maximal surgical safety which can also serve as the basis of a training program.

## Methodology

An expert group of 13 experienced laparoscopic colorectal surgeons, one anatomist, and one graphic artist (and surgeon in training) met three times. In order to achieve our objective of a safe, teachable, and radical CME, the following requirements were initially defined:

- Clear definition and depiction of surgical anatomy.
- Definition of oncologic radicality.
- Description of hazards.
- Drafting of a proposal for a laparoscopic standard technique that provides procedural safety and radicality.
- Inclusion of the critical view concept analogous to that in laparoscopic cholecystectomy (Strasberg: critical view of safety) [17]

Each of the expert surgeons had a minimum of 13 years of post-certification experience (13–30 years) and had performed at least 750 (750–1800) colorectal resections, including > 500 (500–800) laparoscopic colorectal resections and 90–220 laparoscopic right hemicolectomies.

To depict the surgical anatomy, the “open-book” model was further developed from a template published previously [18]. Criteria for the radicality of CME were extracted from a review of the literature.

During the first consensus session, videos of laparoscopic CME right hemicolectomies performed by the experts were individually reviewed by the entire group. Essential or potentially difficult steps of the operation were identified, or their relevance acknowledged for further standardization.

A consecutive workshop evaluated each of the variants and the key steps of the operation which had been consented previously, including the usefulness of the open book model in four body donors. Under the supervision of the anatomist (TW), the operations were performed simultaneously by four rotating teams using the same dissecting and ultrasound-based sealing devices. Port placement was agreed upon on the basis of the most frequent pattern among the participants and applied in all cases (umbilical: 10 mm optic, right lower quadrant: 5 mm; left lower quadrant: 10 mm, left lateral: 5 mm).

After each surgical step, the procedures were interrupted and the results discussed. If there were any complications or critical situations, the supervising anatomist called for a time out and the situation was presented to the entire group. This protocol enabled the surgical expertise of all participants on the progression of the procedures to have maximal effect. It also ensured maximal learning for the whole group from any differences between the body donors (one female, 89 years, obese; two males, 83/84 years, cachectic; one male, 71 years, normal weight). All donors presented with a venous gastro-pancreatico-colic trunk (GPCT) collecting a superior right colic vein (SRCV), a right colic vein (RCV), and a right gastroepiploic vein (RGEV). Since evaluation of CME quality was not the objective of the body donor workshop, no tissue samples were histologically analyzed.

At the end of the workshop, consensus was reached regarding the type and order of the required procedural steps. Each sequence of the operation resulted in representative anatomical scenes. As a result, eight critical views were unanimously adopted as a means of documenting what constituted an adequate operation according to the standardized procedure.

In a third meeting, additional surgical videos were reviewed which documented laparoscopic right hemicolectomies after implementation of the previous consensus. This last step allowed for adjustment and the final definition of the critical views which are described here. Each step and each critical view as well as its sequence within the procedure were discussed until full consensus was reached.

## Results

### Surgical radicality

Target criteria of CME for oncologic radicality:

- Preservation of visceral fascia without injuring the mesocolon.
- Complete removal of the regional lymphatic vessels and lymph nodes.
- Division of the supplying arteries close to their origin (ileocolic artery (ICA), right colic artery, right branch of the middle colic artery).
- Complete removal of the lymphatic tissue along the right side of the superior mesenteric vein (SMV) from approximately 3 cm distal to the ileocolic vein (ICV) up to the GPCT with division of all veins running from the mesocolon into the GPCT (SRCV and RCV).
- Preservation of the GPCT including the pancreatic contributories and the veins that merge into the SMV (e.g., middle colic vein (MCV)).

### Open book model of surgical anatomy

**Fig. 1:**
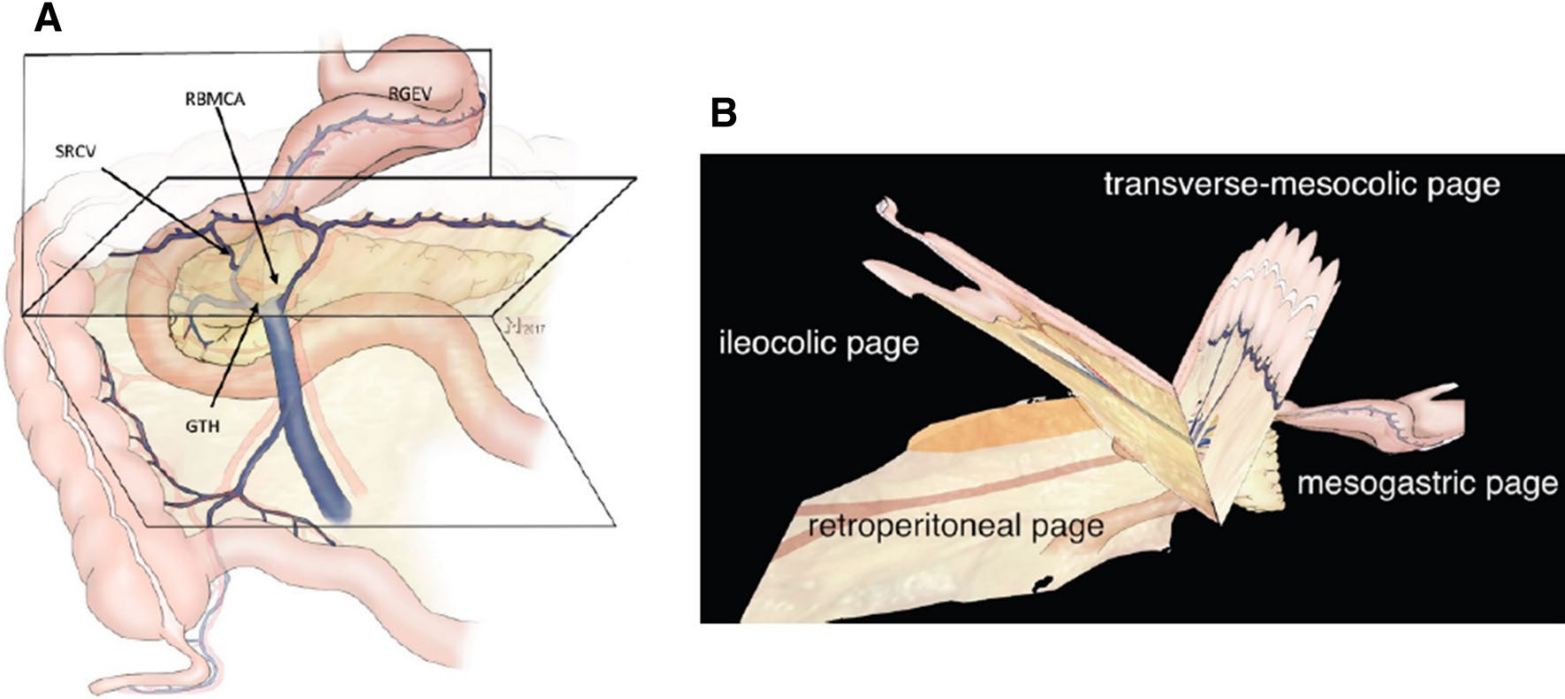
The open book model with the ileocolic-, the transverse mesocolic-, and the mesogastric page is shown (**A, B**). These pages form a symbolic book whose back is located at the axis of the venous GPCT (*SRCV* superior right colic vein, *RBMCA* right branch of middle colic artery, *RGEV* right gastroepiploic vein, *GPCT* gastro-pancreatico-colic trunk of Henle). See also Video No. 1

To facilitate the surgical overview, the open book model of the fascial and vascular relations was developed (Fig. 1, Video No. 1). The “pages” of the book represent the embryologically defined anatomical layers of the involved anatomical structures.

The model allows for a distinction between the extent of resection of standard right hemicolectomy and extended right hemicolectomy. Whereas standard right hemicolectomy is limited to mesocolic excision, extended right hemicolectomy also encompasses partial removal of the mesogastric page with the right gastro-omental vein and the gastro-omental fat body as well as the gastrocolic ligament. The latter is included on the basis of additional passage ways for lymphatic tumor spread in this region [19]. The GPCT, with its inflow from the pancreatic head, is preserved in both standard and extended procedures, unless adjacent lymph nodes are present which can only be removed by sacrificing the GPCT for oncological reasons.

### Standardized operation

The procedure was divided into nine steps, which are individually completed by a critical view of safety [17]. The surgical video examples presented were collected from two separately operated patients by SB and CWS. The duration of surgery was 180 and 187 min, respectively, and both patients had an uneventful course, discharged at postoperative day 5.

#### Step 1

**Fig. 2:**
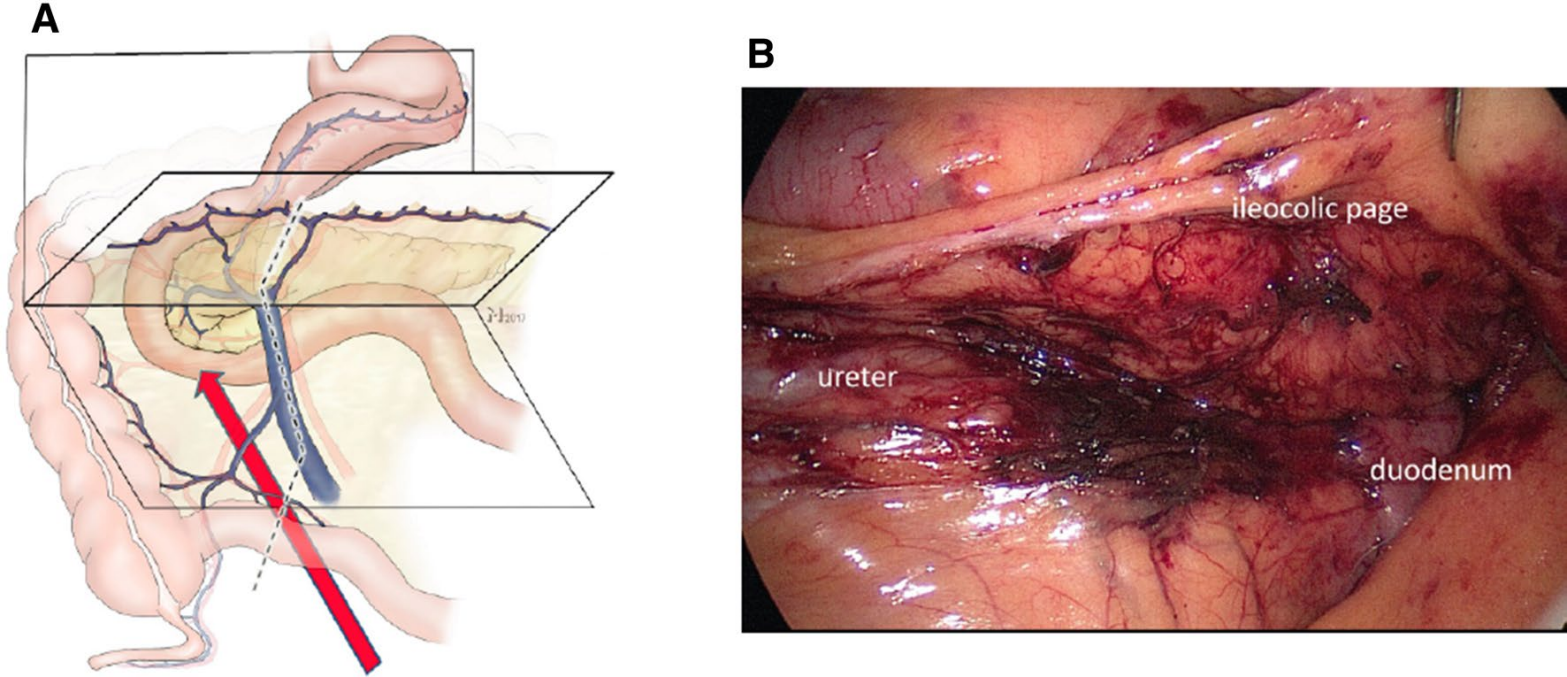
Step 1: the correct dissection plane between the retroperitoneum and the ileocolic page can be ensured when the duodenum is viewed from ventral and below (red arrow) (**A**). The mesenteric root (dotted line) must consequently be located ventral to the viewer´s eye. **B** shows a corresponding intraoperative view (see also Video No. 2) with the duodenum and pancreas dorsal and the mesocolon ventral to the level of dissection. This view from medial under the ileocolic page is established by the medial to lateral dissection approach exposing the retroperitoneum. (Color figure online)

The dorsal aspect of the ascending mesocolon and the mesenteric root are mobilized while preserving the lateral colonic attachments. This can either be performed starting from the duodeno-jejunal flexure, uncinate first approach [12], or from the distal mesoilium in a medial to lateral fashion [11] (Fig. 2, Video No. 2, surgeries by SB and CWS).

##### Critical view 1

View caudally showing the dorsal aspect of the mesenteric root, the third part of the duodenum, and the uncinate process (Fig. 2A, B, Video No. 2).

#### Step 2

Identification of the junction between the ileocolic and the superior mesenteric vessels in order to avoid inadvertent incision of the mesentery on the left side of the SMV which can result in loss of orientation and injury of the SMV and SMA. The vessels can be identified as a V shaped configuration which can be facilitated by traction to the appendix.

##### Critical view 2 (V-View)

**Fig. 3:**
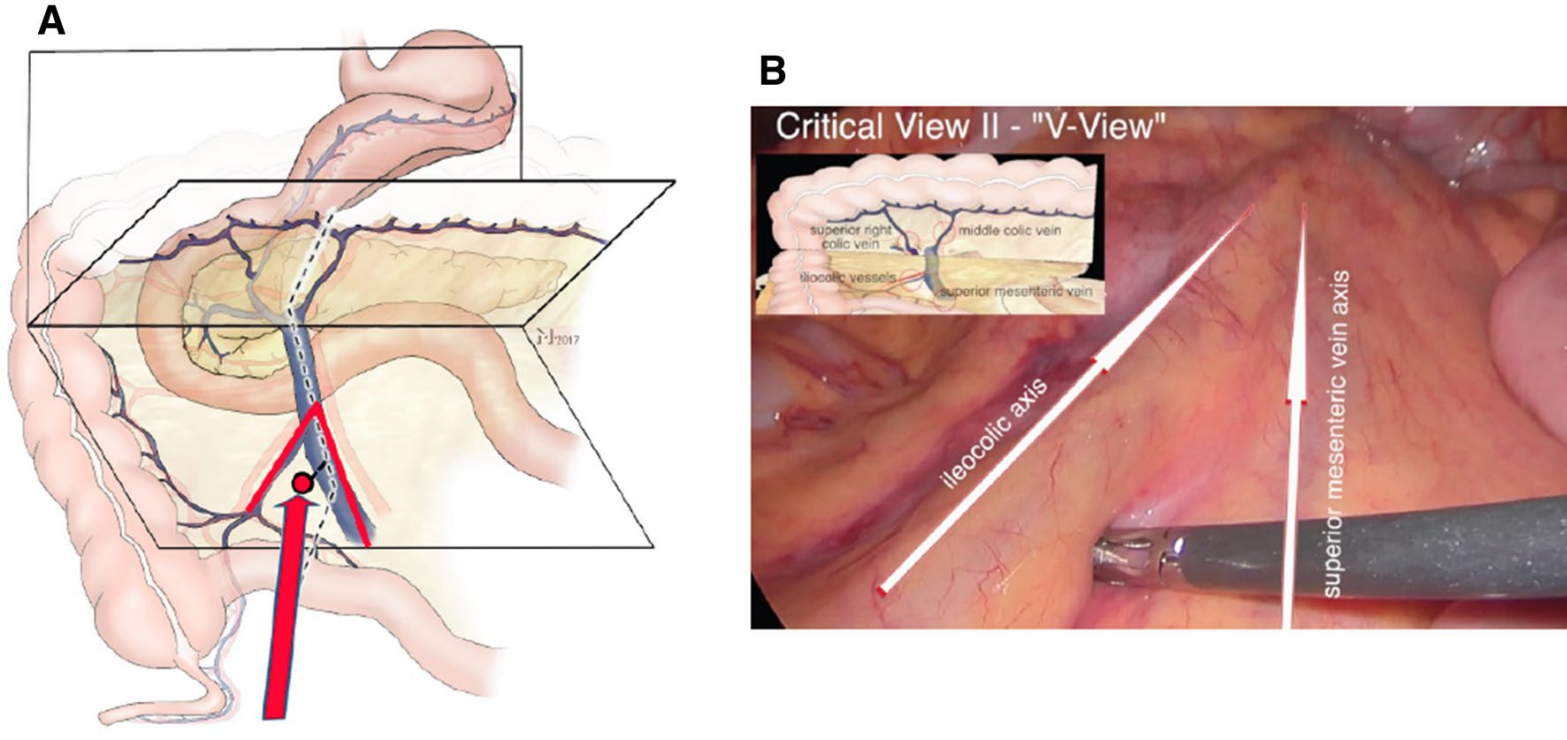
Step 2: the V-View determines (**A**) the area of the ileocolic mesentery distal to the ileocolic vessels and on the right side of the SMV. **B** Shows the corresponding intraoperative view (Video No. 2) with the ileocolic and SMV axis highlighted (arrows)

Ventral aspect of the mesentery from a caudal perspective showing the V-shaped confluence of the ileocolic and the SMV/SMA vessels (Fig. 3, Video No. 2).

#### Step 3

The mesentery is entered between the ileocolic- and the superior mesenteric vessels with app. 3 cm distance to the confluence, which results in a window to the dorsal dissection plane (step 1). The SMV can now be followed up to the confluence of the ICV and the SMV. This ensures that the lymphatics at the ileo-mesenteric junctions are completely resected. This approach also increases safety because the control of the SMV is facilitated due to its dorsal mobility. Before division of the ICV, the confluence (SMV/ICV) is completely dissected.

##### Critical view 3

An instrument passed behind the ICV ensures unequivocal circular dissection.

#### Step 4

**Fig. 4:**
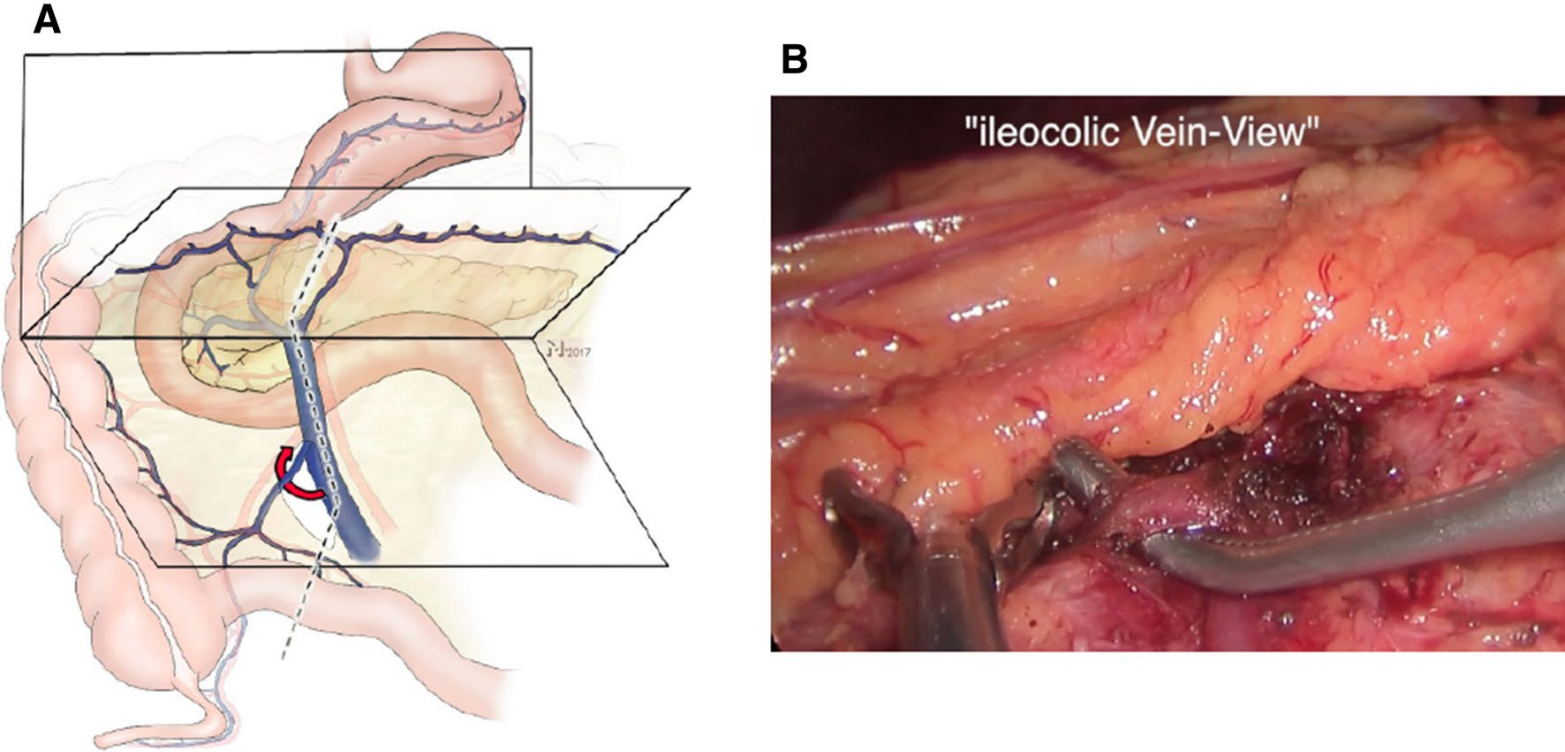
Step 3: **A** after incision of the ileocolic mesentery the SMV is dissected and the ICV divided (curved red arrow). **B** Shows the corresponding intraoperative view (Video No. 2) with the ICV after dissection. (Color figure online)

**Fig. 5:**
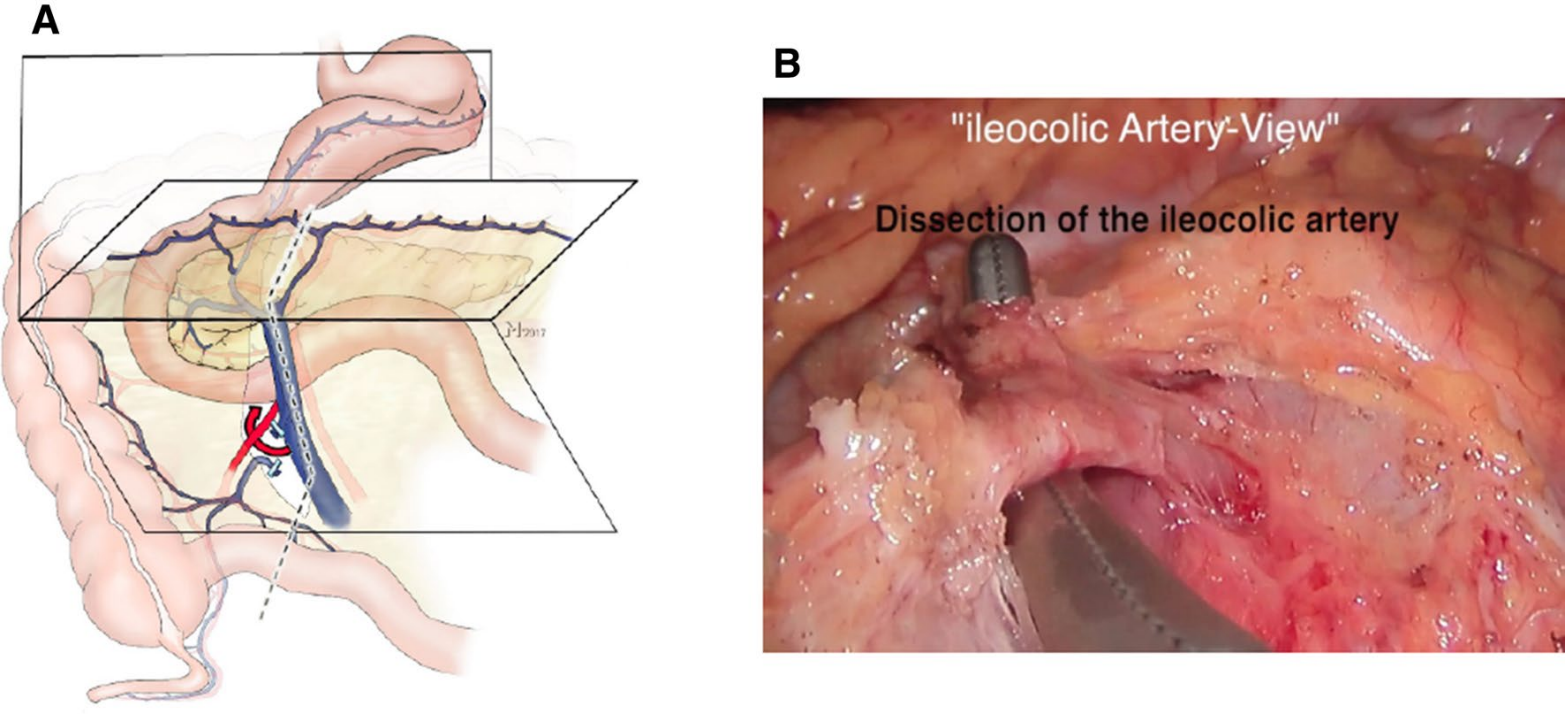
Step 4: **A** division of the ICA, **B** shows the corresponding intraoperative view (Video No. 2) with the ICA before division (curved red arrow). The preparation then continues along the dotted line (**A**) in the axis of the SMV. (Color figure online)

In this step, the ICA is divided. If the artery runs anterior to the SMV, it is divided close to its origin at the SMA. If the ICA crosses dorsal to the SMV, its division is performed at the level of the right border of the SMV. Using this approach, often the SMA becomes visible dorsal to the SMV so that a complete lymphadenectomy on the right aspect of the SMA can be performed (Figs. 4, 5, Video No. 2).

##### Critical view 4

An instrument is passed behind the ICA.

The order of the steps 3 and 4 is sometimes altered depending on the course of the ICA (Video No. 2).

#### Step 5

Further cephalad preparation along the SMV is terminated at the level of the inflow of the GPCT. Then the lesser sac is opened while preserving the gastro-omental arcade.

##### Critical view 5

**Fig. 6:**
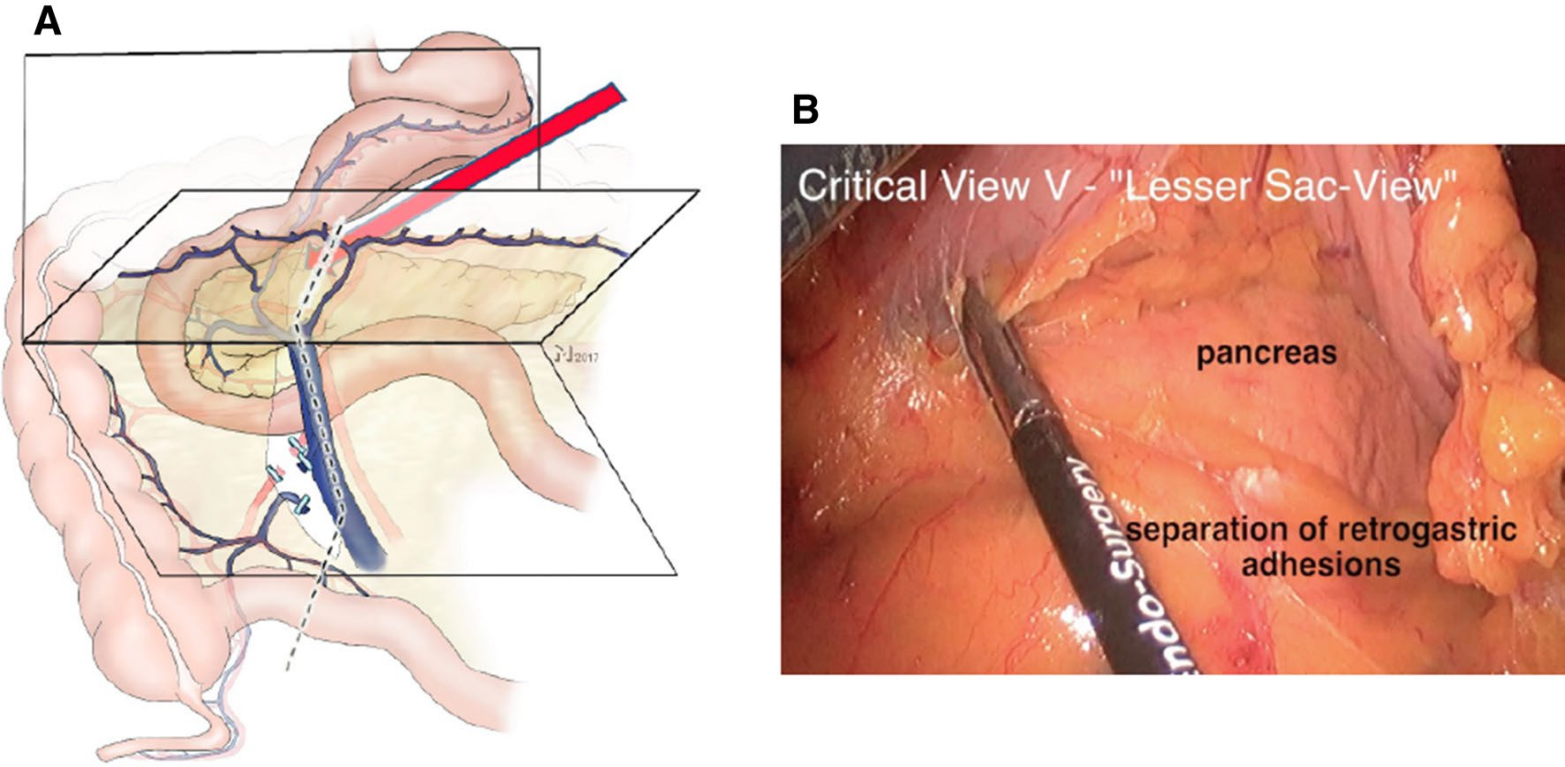
Step 5: before the dissection line on the right side of the middle colic vessels can be followed toward the transverse colon, the lesser sac needs to be opened by entering the gastrocolic ligament from the left side (red arrow **A**), which allows for a two-sided approach of the transverse mesocolic mesentery and the venous confluence of the GPCT. **B** Shows the corresponding intraoperative view (Video No. 3) with the lesser sac opened and the pancreas visible. (Color figure online)

View into the lesser sac on the posterior wall of the stomach (Fig. 6, Video No. 3, CWS).

#### Step 6

The division of the gastrocolic ligament is continued until the hepatic flexure is reached while separating the transverse mesocolon from the dorsal mesogastrium. In the posterior direction, this separation ends where the colic veins (RCV, SRCV) merge with the GPCT. Dissection step 6 results in a sulcus running from the area of the middle colic vessels to the anterior aspect of the duodenum (part II) between the cephalad mesogastrium and the caudally located transverse mesocolon. The GPCT and—further to the left— the pancreatic body, mark the posterior border of the sulcus. In relation to the sulcus, the course of the SRCV is special in the respect that it bridges the gap between the transverse mesocolic and the mesogastric page in the open book model before it merges into the GPCT (Video No. 1). Therefore, dissection of the SRCV needs to avoid inappropriate tension.

##### Critical view 6 (lesser sac sulcus view)

**Fig. 7:**
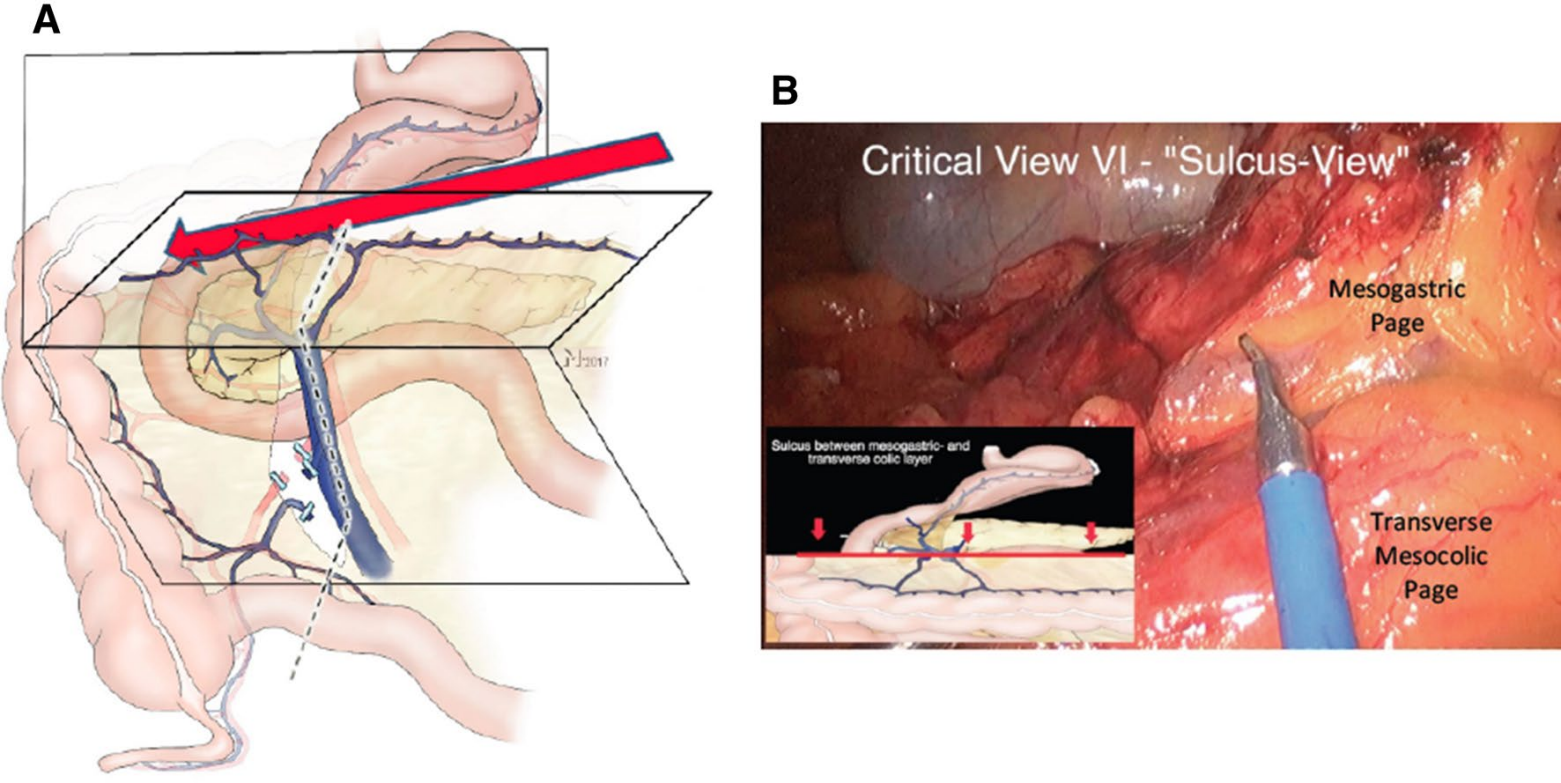
Step 6: dissection following the transverse mesocolon superiorly leads to the establishment of a sulcus (**A**). This sulcus runs along the inferior border of the pancreas, reaches the venous GPCT on the right side of the SMV, and crosses the pancreatic head toward the ventral renal fascia. The SRCV requires special attention as it bridges the gap between the transverse mesocolic and the mesogastric page. **B** Shows the corresponding intraoperative view (Video No. 3) with the lesser sac opened and the pancreas visible

View from medial to lateral shows the sulcus between the mesogastrium and transverse mesocolon on the pancreatic head crossing over the duodenum. The anterior aspects of the SRCV and the right gastro-omental veins can be identified (Fig. 7, Video No. 3).

#### Step 7

Before division of the right MCA branches, perfusion of the left sided colon has to be ensured. If the branches of the MCA cannot be identified prior to incision of the transverse mesocolon, it is opened strictly anterior to the SMV where the artery and its branches can be found. The MCV is preserved.

##### Critical view 7

**Fig. 8:**
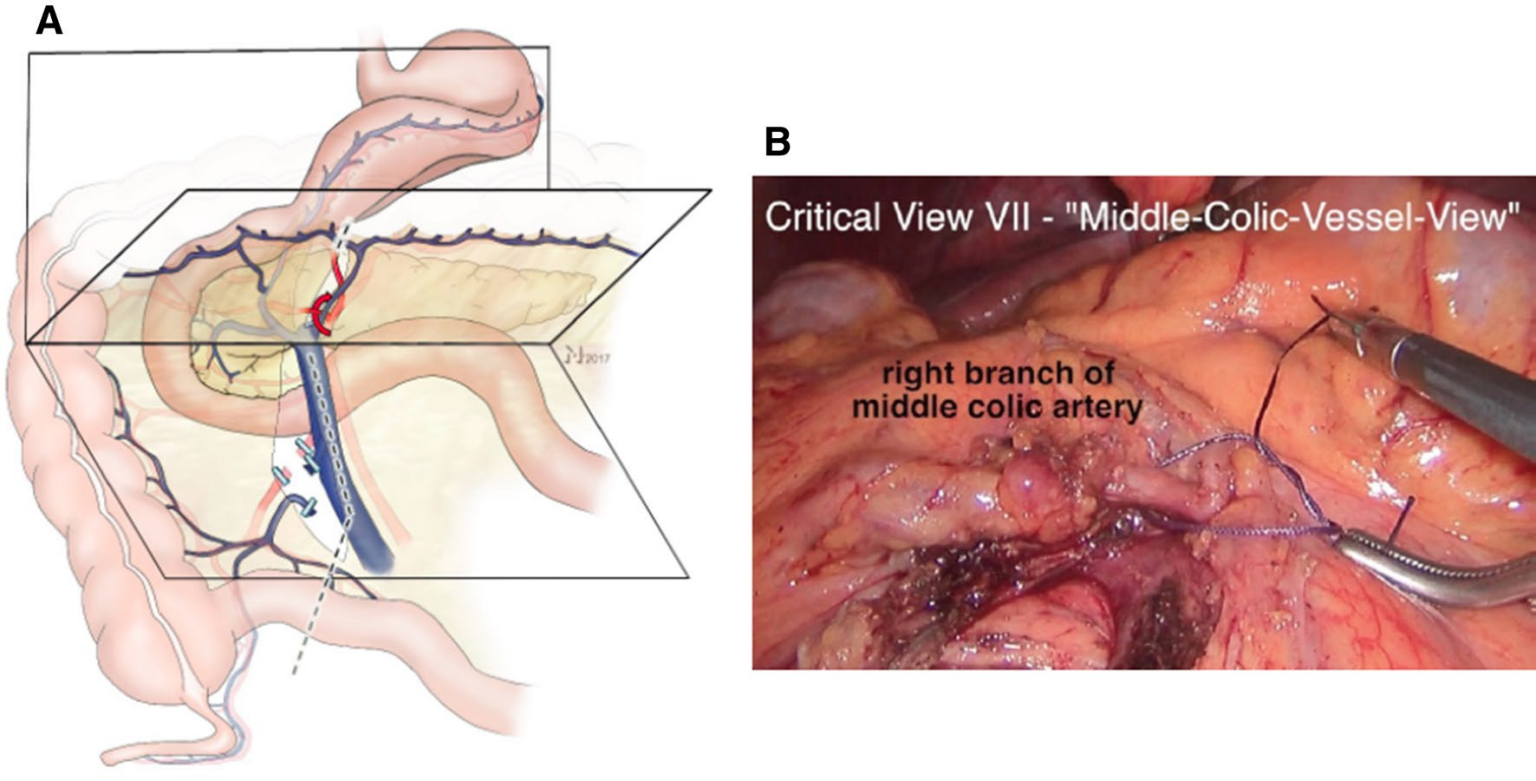
Step 7: division of the transverse mesocolic mesentery must not compromise left transverse mesocolic perfusion. This can only be ensured when division of arterial vasculature is limited to the right branch of the middle colic artery (**A**) which has to be unmistakably identified (curved red arrow). **B** Shows the corresponding intraoperative view (Video No. 4) with the right branch of the middle colic artery encircled with a ligature

An instrument is passed behind the right branch of the MCA close to its origin demonstrating intact arterial supply to the left transverse colon (Fig. 8, Video No. 4, SB).

#### Step 8

At this stage, the only vascular attachments of the mesocolon are the veins that run into the GPCT. The consented strategy was to dissect the anterior aspect of the GPCT and divide all colic veins that enter it anteriorly. It is of note that the GPCT itself and the pancreatic veins are neither encircled nor divided, which stands in contrast to other reports [13]. The right gastro-omental vein is also preserved, unless for extended right hemicolectomy.

##### Critical view 8

**Fig. 9:**
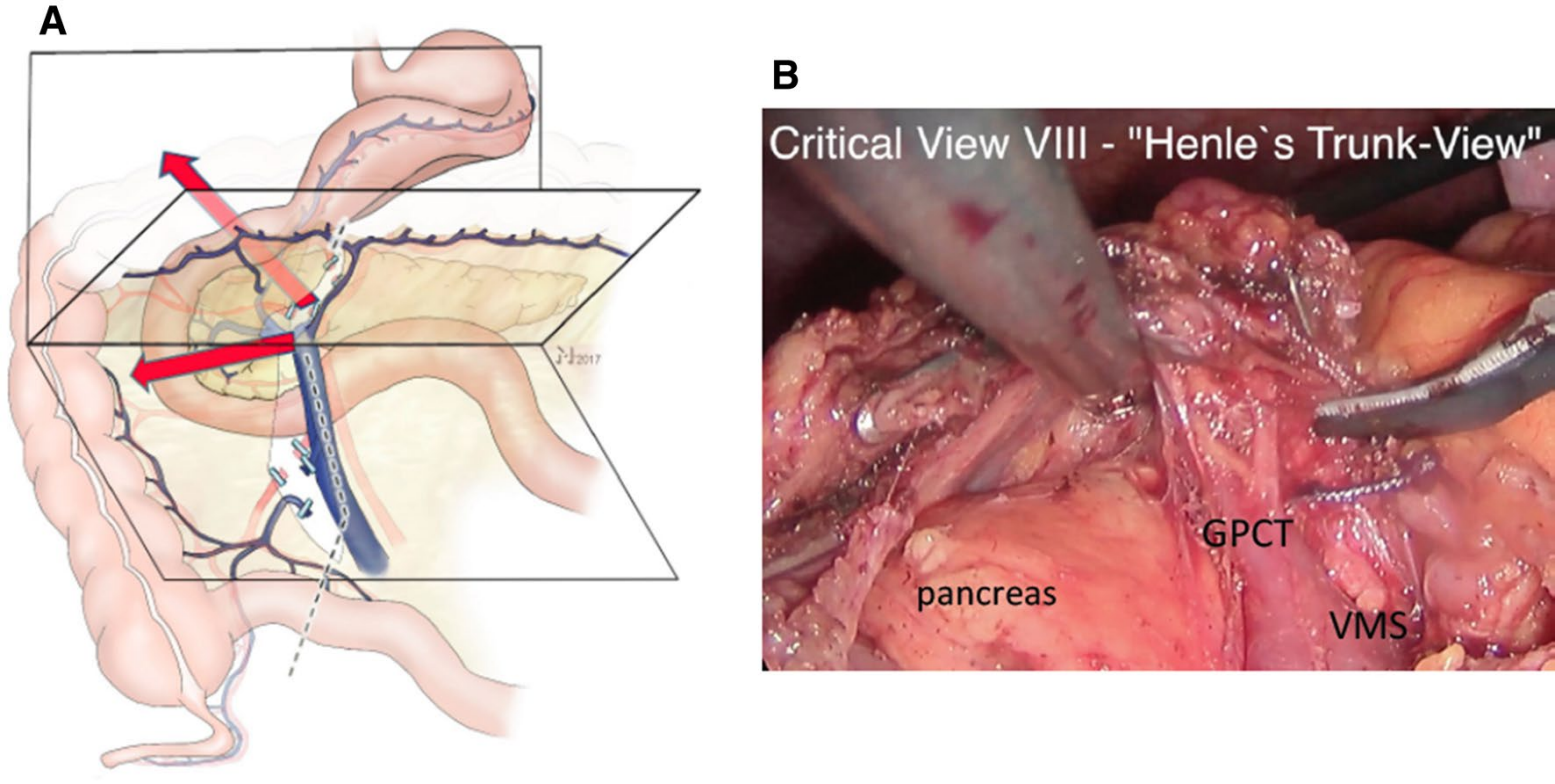
Step 8: the dissection of the venous confluence of the GPCT completes the mobilization of the colonic mesentery prior to bowl resection. Ventrally adjacent lymphatic tissue to the GPCT together with the SRCV remains with the resected specimen to ensure oncologic radicality. The trunk itself remains in situ (**A**). Furthermore, complete control of this vessel region prevents the risk of bleeding due to avulsion of the vessel when the colon is exteriorized from the abdomen for resection and anastomosis. **B** Shows the corresponding intraoperative view (Video No. 4) shortly before division of the GPCT veins

Anterior aspect of the intact venous GPCT of Henle, GPCT-view (Fig. 9, Video No. 4).

#### Step 9

This step completes the operation with division of the lateral attachments of the ascending colon. Whether an extra- or intracorporeal resection and anastomosis are performed was left to the discretion of the surgeon.

## Discussion

According to current evidence, right hemicolectomy with CME for colon cancer offers a 10% additional advantage for 4-year disease-free survival [5, 6, 20]. Criteria for surgical radicality used in these reports were comparable to the ones consented in our group [2]. Because the procedure is more complex than traditional hemicolectomy, it increases the risk for severe complications. This is especially true for laparoscopic right hemicolectomy. Consequently, if the oncological advantage of CME is not to be jeopardized and the advantages of the laparoscopic approach [21–23] are to be maintained, a systematic training of colorectal surgeons is necessary for routine implementation of this method. A prerequisite of such training is a consensus about the relevant surgical anatomy and about how the operation should be performed. Here, we present a suggestion that addresses both aspects of these consensus requirements.

In a right hemicolectomy, the complexity of the anatomy is related to the mesenteric planes and to the vascular anatomy. Both are visualized in the open book model, in respect to the requirements of laparoscopic hemicolectomy. This model will support surgical orientation and it facilitates communication between trainer and trainee. In this way, the open book model helped to clarify controversial discussions among the authors and it also improved their initial teaching experiences.

Much of the consensus discussion was related to defining surgical steps that minimize inadvertent tissue injury and facilitate the management of intraoperative complications, for example, by improving accessibility to critical vascular structures. This allowed the dissection of the planes on both sides of the vessels (as one opens the pages of a book) to be performed before division of the vessels. This is a clear difference to other techniques of laparoscopic hemicolectomy—including the classical medial approach. For example, when the medial approach is applied, the ileocolic vessels and the SMV are dissected before the mesenteric root and the mesentery of the ascending colon are mobilized. Under these circumstances, injuries to the SMV or the ileocolic vessels are hard to control. The same holds true for the middle colic vessels and the veins joining the GPCT. Control of the GPCT can only be reliably achieved when the space between the mesogastric and the transverse mesocolic page are separated and the SMV has been unmistakably dissected.

The operation was divided into well-defined steps in order to facilitate adherence to the suggested sequence. In addition, the concept of the critical view of safety was adopted from laparoscopic cholecystectomy. Because laparoscopic right hemicolectomy is more complex, eight specific critical views were introduced. Failure to achieve the critical views should prompt the surgeon to re-evaluate the anatomy and consider conversion to laparotomy, so that safety and radicality are not compromised. This is analogous to the widely held view that, when Strassberg’s view of safety in laparoscopic cholecystectomy is not achieved, conversion may be indicated.

An additional focus of the consensus process was to choose critical views that are independent of surgical devices and dissection techniques. As a result, port placement and patient positioning, choice of sealing devices, or even robot use or the location of the mini laparotomy are not in the scope of standardization. In this respect, our suggestion should be considered as the description of a surgical principle rather than a text-book guidance that can be applied independently of operation room setting and choice of surgical instruments.

The strength of our work is that a large group of experienced surgeons together with an anatomist were able to find a consensus about a putative safe way to perform and teach laparoscopic right hemicolectomy with CME.

However, our expert opinion alone does not constitute evidence that the standardized proposed procedure is safer in practice than any other approach to laparoscopic right hemicolectomy with CME. Therefore, our group has initiated a multicenter trial (DRKS-ID: DRKS00012369) in which the procedure is evaluated. The first patient was recruited in July 2017.

In conclusion, the suggested standardized approach for laparoscopic right hemicolectomy with CME based on the open book model and using the critical views of safety may contribute to a safe implementation of this procedure in routine practice.

## Electronic supplementary material

Below is the link to the electronic supplementary material.

Supplementary Video No. 1 (M4V 72322 KB)

Supplementary Video No. 2 (M4V 49255 KB)

Supplementary Video No. 3 (M4V 46467 KB)

Supplementary Video No. 4 (M4V 50871 KB)
